# Supplementary material for: TmPGRP-SA regulates Antimicrobial Response to Bacteria and Fungi in the Fat Body and Gut of Tenebrio molitor
Source: Int J Mol Sci. 2020 Mar 19;21(6):2113. doi: 10.3390/ijms21062113 (PMC7139795; doi:10.3390/ijms21062113)
Supplement: Supplementary file 1 [file ijms-21-02113-s001.pdf]

International Journal of
*Molecular Sciences*

MDPI

# Supplementary

```
>TmPGRP-SA
TACGGCCGGGGATTTCCAAGTTGCATCCGATCAGAGGACATCATAACATAAAGTCTGCAT
AAAGTAAAACAGTGAATAGGTCACATTTGTCGTTCAACTAACTGCTAGATATTGTTGTTT
TGTTACA
>>>>>>>>>>>TmPGRP-SA_FW_Cloning
ATGCACGGAGTGTTGTGTTTTTTATTAAGTTTTGTGCGTGTTTATCAAATTTCGGCTCTC
TCAGGTTCTACGATACCAAGAATATGTCCTGAAATTATTAGTCGGACAAGATGGGGGGCG
AGAACTCCATTAGAAGTGGATTATTCATTAATTCCCATTGAAAATGTCGTTGTTCATCAT
                 >>>>>>>>>>>>>TmPGRP-SA_Fw_dsRNA
ACTGTAACTCATACATGCGACTCGGAAAGCGAATGTGCAACTCTTTTGAGAAATGTTCAG
AATTTTCACATGGAAAACTTAGAATTTCATGACATAGGATACAACTTTTTGGTTGCAGGT
GACGGACAAATATACGAAGGAGCGGGTTGGCATAAAGTTGGAGCGCATACCAGAGGCTAC
AATACAAGATCCTTGGGATTAGCCTTTATTGGCAACTTCACAAGCCAACTACCAGTCCAA
AAACAGCTTAAAGTTGCTAAAGATTTTCTTCAATGCGGAGTTGAACTGGGAGAATTAAGT
                   TmPGRP-SA_REV_dsRNA<<<<<<<<<<<<<
                   TmPGRP-SA_FW_qPCR>>>>>>>>>>>>>>>
AAAAATTATAAATTATTTGGAGCACGCCAAGTGAGTTCGACAAGCAGCCCTGGACTGAAA
                       TmPGRP-SA_REV_qPCR<<<<<<<<<<<<<<<<<
CTCTACCGTGAACTGCAAGATTGGCCCCATTTCACCAGATCTCCTCCTAAATAA
<<<<<                   TmPGRP-SA_REV_Cloning<<<<<<<<<<<<<<<<
ATTCATCAATTGTTCAACAATTGTTGTATTTTATATTATTGTTTTATGTCATTAAATAAT
TGAATTATCG

MHGVLCFLLSFVRVYQISALSGSTIPRICPEIISRTRWGARTPLEVDYSLIPIENVVVHH
TVTHTCDSESECATLLRNVQNFHMENLEFHDIGYNFLVAGDGQIYEGAGWHKVGAHTRGY
NTRSLGLAFIGNFTSQLPVQKQLKVAKDFLQCGVELGELSKNYKLFGARQVSSTSSPGLK
LYRELQDWPHFTRSPPK*
```

**Figure S1.** The full length cDNA sequence of *TmPGRP-SA.* The position of all primers used for real-time analysis and dsRNA synthesis are indicated. The cloning primer sequences are presented in green, qPCR primer sequences in blue, and double-stranded RNA primer sequences in red.

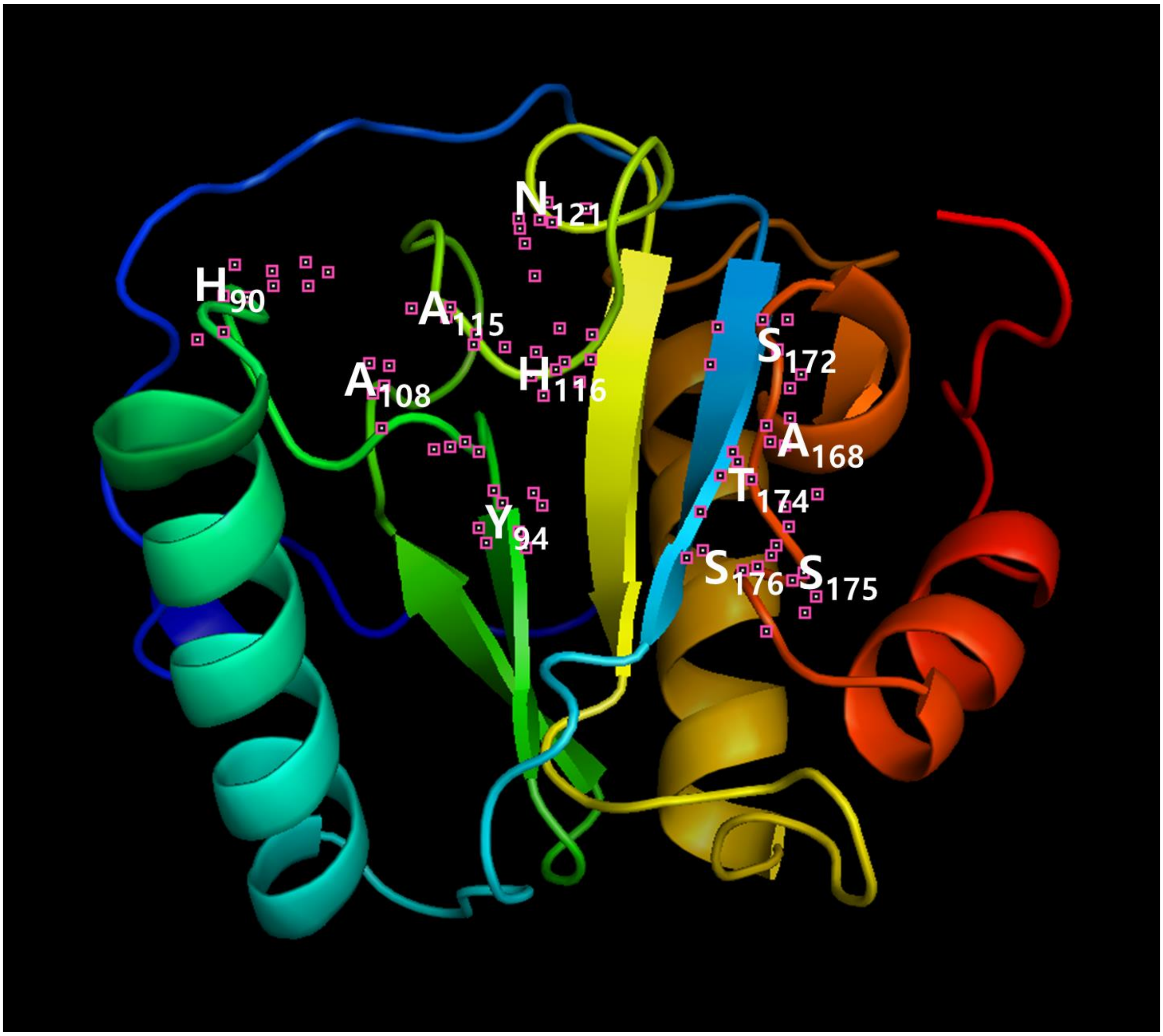


**Figure S2.** 3D structure of *Tm*PGRP-SA. SWISS-MODEL and PyMOL visualized the amidase catalytic site (active site) and chemical (substrate) binding site amino acid residues (N-terminal: blue, C-terminal: red). The conserved amino acids are shown in small pink squares and name of the amino acids is represented in white color.

| | *Tc*PGRP-SA | *Lm*PGRP-SA | *Gb*PGRP-SA | *Bc*PGRP-SA | *Bk*PGRP-SA | *Bi*PGRP-SA | *Ds*PGRP-SA | *Dm*PGRP-SA | *Db*PGRP-SA | *Bl*PGRP-SA | *Zc*PGRP-SA | *Hs*PGLYRP3 | *Ej*PGRP-SA |
|---|---|---|---|---|---|---|---|---|---|---|---|---|---|
| *Tm*PGRP-SA | 76 | 50 | 43 | 37 | 37 | 38 | 43 | 44 | 43 | 43 | 45 | 38 | 27 |
| *Tc*PGRP-SA | | 52 | 42 | 40 | 40 | 41 | 45 | 46 | 42 | 42 | 44 | 38 | 26 |
| *Lm*PGRP-SA | | | 56 | 42 | 42 | 42 | 49 | 49 | 49 | 47 | 48 | 43 | 26 |
| *Gb*PGRP-SA | | | | 44 | 44 | 47 | 43 | 42 | 41 | 44 | 44 | 36 | 25 |
| *Bc*PGRP-SA | | | | | 100 | 91 | 40 | 41 | 38 | 42 | 43 | 36 | 23 |
| *Bk*PGRP-SA | | | | | | 91 | 40 | 41 | 38 | 42 | 43 | 36 | 23 |
| *Bi*PGRP-SA | | | | | | | 41 | 41 | 39 | 42 | 43 | 36 | 21 |
| *Ds*PGRP-SA | | | | | | | | 98 | 69 | 66 | 66 | 38 | 31 |
| *Dm*PGRP-SA | | | | | | | | | 68 | 66 | 66 | 38 | 31 |
| *Db*PGRP-SA | | | | | | | | | | 69 | 69 | 35 | 27 |
| *Bl*PGRP-SA | | | | | | | | | | | 91 | 38 | 26 |
| *Zc*PGRP-SA | | | | | | | | | | | | 38 | 26 |
| *Hs*PGLYRP3 | | | | | | | | | | | | | 26 |
| *Ej*PGRP-SA | | | | | | | | | | | | | |

% Identity

**Figure S3.** Percent identity with full-length ORF of *TmPGRP-SA* and other insects.

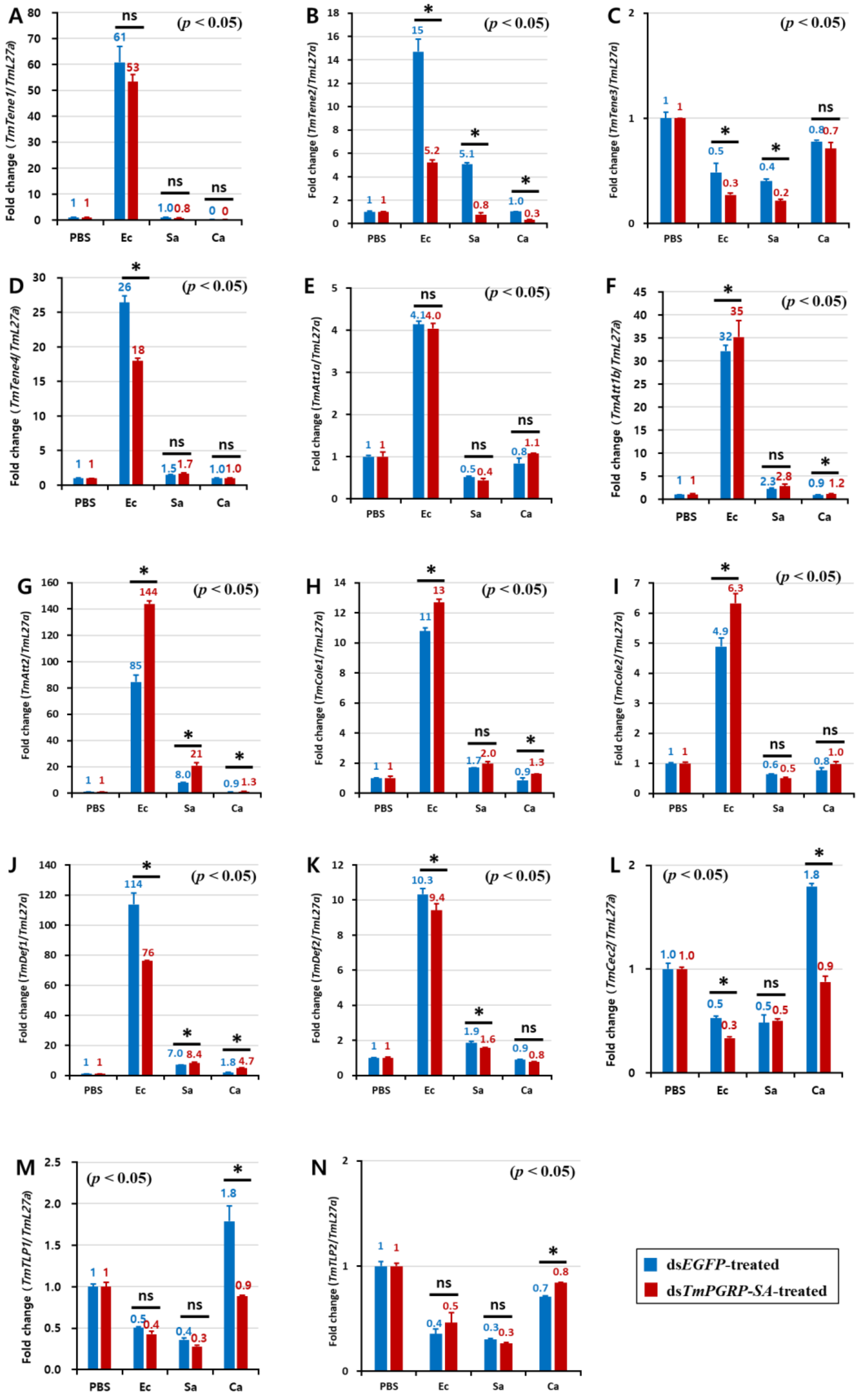


**Figure S4.** AMP expression levels in the *TmPGRP-SA*-knockdown *T. molitor* larval hemocytes upon *E. coli* (Ec), *S. aureus* (Sa), and *C. albicans* (Ca) infections. Healthy larvae (10th–12th instar) were injected with ds*TmPGRP-SA* and infected with a suspension of *E. coli*, *S. aureus*, or *C. albicans* on the second day post-dsRNA injection. PBS-injected larvae were used as controls. The expression level of the AMP genes *TmTenecin-1* (**A**), *TmTenecin-2* (**B**), *TmTenecin-3* (**C**), *TmTenecin-4* (**D**), *TmAttacin1a* (**E**),

*TmAttacin-1b* (**F**), *TmAttacin-2* (**G**), *TmColeoptericin-1* (**H**), *TmColeoptericin-2* (**I**), *TmDefensin1* (**J**), *TmDefensin2* (**K**), *TmCecropin-2* (**L**), *TmThaumatin-like protein-1* (**M**), and *TmThaumatin-like protein-2* (**N**) were measured using qRT-PCR and compared with the ds*EGFP* treated groups. ds*EGFP* was used as negative control and *TmL27a* as an internal control. The numbers above the bars indicate AMP expression levels. All experiments were repeated three times with similar results. Statistical analysis was performed using Student's t-tests (* $p < 0.05$) and ns: no significant.
